# Magnetisation transfer as a biomarker for chronic airway fibrosis in a mouse lung transplantation model

**DOI:** 10.1186/s41747-017-0032-3

**Published:** 2018-02-12

**Authors:** David Kenkel, Yoshito Yamada, Markus Weiger, Moritz C. Wurnig, Wolfgang Jungraithmayr, Andreas Boss

**Affiliations:** 1 Department of Diagnostic and Interventional Radiology, University Hospital Zurich, Ramistrasse 100, 8091 Zurich, Switzerland; 2 Division of Thoracic Surgery and Department, University Hospital Zurich, Zurich, Switzerland; 3 Institute for Biomedical Engineering, University and ETH Zurich, Zurich, Switzerland; 4 Department of Thoracic Surgery, Medical University Brandenburg, Neuruppin, Brandenburg Germany

**Keywords:** Chronic airway fibrosis (CAF), Chronic Lung allograft dysfunction (CLAD), Magnetisation transfer ratio (MTR), Mouse animal model, Zero echo time (ZTE) imaging

## Abstract

**Background:**

Chronic airway fibrosis (CAF) is the most prevalent complication in human lung transplant recipients. The aim of the study is to evaluate magnetisation transfer (MT) as a biomarker of developing CAF of lung transplants in a mouse model.

**Methods:**

Lung transplantation was performed in 48 mice, applying major or minor histocompatibility mismatches between strains for the induction of CAF. MT measurements were performed in vivo with systematic variation of off-resonance frequencies and flip angle of the MT prepulse. MT ratios (MTRs) were compared for lungs showing CAF and without CAF.

**Results:**

Seven out of 24 animals (29%) showed a pattern of CAF at histology. All mice developing CAF also showed signs of acute rejection, whereas none of the lungs showed signs of other post-transplant complications. After lung transplantation, pulmonary infiltration was a frequent finding (14 out of 24) exhibiting a higher MTR (24.8% ± 4.5%) compared to well-ventilated lungs (12.3% ± 6.9%, *p* = 0.001) at 8000 Hz off-resonance frequency, 3000° flip angle. In infiltrated lung tissue exhibiting CAF, lower MTR values (21.8% ± 5.7%) were found compared to infiltrated lungs showing signs of acute rejection alone (26.5% ± 2.9%, *p* = 0.028), at 8000 Hz, 3000° flip angle. The highest MTR values were observed at 3000° flip angle, using a 1000 Hz off-resonance frequency.

**Conclusion:**

MTR might serve as a tool for the detection of CAF in infiltrated lung tissue.

## Key points

- Chronic airway fibrosis (CAF) is a complication seen in lung transplant recipients.
- The meaning of magnetisation transfer (MT) in lung diseases has, so far, been poorly investigated.
- In infiltrated lung tissue exhibiting CAF, lower MT ratio values were found in this preclinical study on transplanted syngeneic mice.
- MT might serve as a tool for the detection of CAF in infiltrated lung tissue.

## Background

For many patients with end-stage pulmonary disease, lung transplantation is the last therapeutic option. However, major problems are long-term complications such as chronic lung allograft dysfunction including bronchiolitis obliterans syndrome and restrictive allograft syndrome [1–3]. Bronchiolitis obliterans syndrome develops in 35–60% of the recipients and is a major limiting factor for long-term survival [4]. Bronchiolitis obliterans syndrome is histologically defined by a thickening of the walls of the airways caused by migration of fibroblasts, myofibroblasts and lymphocytes, and the resulting synthesis of collagen [5]. It is clinically defined as a restrictive and obstructive impairment in the absence of infection. In pulmonary function testing, a decrease > 20% in the forced expiratory volume in 1 s compared to the postoperative baseline value is considered to be diagnostic for bronchiolitis obliterans syndrome [1, 4, 6, 7]. Restrictive allograft syndrome is defined by pleuroparenchymal fibroelastosis different from bronchiolitis obliterans syndrome associated with a diffuse alveolar damage [2]. After all, either phenotype of chronic lung allograft dysfunction is regarded as chronic airway fibrosis (CAF).

Computed tomography is the current standard technique for the postoperative evaluation of the transplanted lung parenchyma due to its non-invasiveness compared to biopsy. However, findings in CAF are not specific and can also be seen in other pulmonary complications after lung transplantation. These non-specific image manifestations include peribronchial cuffing, subsegmental atelectasis and air trapping [1, 6]. Currently, there is no imaging modality that enables the early and specific detection of CAF, thereby precluding the optimal treatment in this setting.

Advances in magnetic resonance imaging (MRI) techniques with the development of radial k-space sampling lead to the possibility of depicting lung parenchyma at high field strength, a capability which is, however, hampered by the low spin-density and the rapid signal decay caused by microscopic magnetic field inhomogeneities generated by tissue–air interfaces [8–12]. In view of recent technological advances, the role of MRI in the depiction of lung diseases can be revisited and further investigated. In particular, magnetisation transfer (MT) with its property to depict and quantify changes in tissue macromolecular composition is a promising approach [13–17]. Therefore, the goal of our study was to evaluate the role of MT as a possible biomarker for the detection of CAF in mice with chronic lung allograft dysfunction.

## Methods

### Animals and transplantation procedure

Syngeneic mice (*n* = 48) were used in this study, including 24 mice serving as lung transplant donors and 24 mice serving as recipients. The weight of the mice was in the range of 25–30 g. The transplantation procedure was carried out as originally described [18–20]: all mice received general anaesthesia; laparotosternotomy was performed in donor mice to explant the graft; thereon lung grafts were stored in a preservation solution at 4 °C until transplant.

Two different levels of mismatch in the major histocompatibility complex (MHC) combinations of the donor and the grafted mouse and two different time points after the explantation of the graft were evaluated, because the most conclusive protocol to develop CAF in mice had not been introduced yet. A minor MHC mismatch model (*n* = 19) was implemented utilising either: (1) C57BL/10 mice as donors and C57BL/6 mice as recipients (*n* = 17 pairs); or (2) C57BL/6 as donors and C57BL/10 as recipients (*n* = 2 pairs). The combination of C57BL/6 as donors and C57BL/10 as recipients was explanted eight weeks after transplantation (*n* = 2). The combination of C57BL/10 as donors and C57BL/6 as recipients was explanted either eight (*n* = 10) or 12 (*n* = 7) weeks after transplantation. The combination of BALB/c as donor and C57BL/6 as recipient was used as a major MHC mismatch model (*n* = 5), These transplants were explanted eight weeks after transplantation and immediately evaluated. Table 1 summarises the different mismatch combination types and the time intervals between transplantation and explantation. MRI measurements were performed on the day of the explantation procedure.

**Table 1:** Summary of the different MHC mismatch combination types, time intervals between transplantation and MRI examination/tissue explantation, number of CAF cases examined for each mice group and mean acute rejection scores in the adopted experimental settings

Type of MHC mismatch combination	Time between transplantation and explantation (weeks)	Chronic airway fibrosis (n)/animals examined (n)	Acute rejection score based on ISHLT guidelines^a^ of all CAF and no-CAF mice (A1–A4, mean ± SD)
Major (D:BALB/c; R:C57BL/6)	8	3/5	3.80 ± 0.27
Minor (D:C57BL/10; R:C57BL/6)	8	2/10	1.73 ± 1.38
Minor (D:C57BL/10; R:C57BL/6)	12	1/7	2.71 ± 0.95
Minor (D:C57BL/6; R:C57BL/10)	8	1/2	2.75 ± 1.06

^a^ISHLT guidelines [3]

*D* donor, *R* recipient

In accordance with *The Principles of Laboratory Animal Care* (most recently revised in 1996 [21]), care of the animals was taken, e.g. regarding adaptation and housing conditions. The study was approved by the local veterinary office.

### Histologic examination

After MRI, animals were euthanised and the graft and the native lung were explanted, formalin fixated, paraffin embedded and sectioned. The sections were prepared using Masson’s trichrome stain for the depiction of collagen [22]. We defined CAF as eosinophilic hyaline fibrosis in the sub-mucosa of membranous and respiratory bronchioles or intraluminal fibrosis of the airways. Further sections were stained with haematoxylin and eosin allowing the depiction of other post-transplant complications such as acute rejection. The sections were evaluated according to the criteria of the International Society of Heart and Lung Transplantation (ISHLT) by blinded reading; acute rejection pathology was graded using the standard criteria of the ISHLT for clinical lung transplantation [3].

### MRI

A 4.7-T PharmaScan animal MR scanner (Bruker, Ettlingen, Germany) was used. The mice were scanned head-first in the prone position and placed centrally in the linear polarised hydrogen whole-body mouse radiofrequency coil. The animal bed was equipped with a pad with continuous warm water supply to avoid cooling. The mice were anesthetised with isoflurane during image acquisition. The concentration for the induction of the anaesthesia was 5%/l and for the maintenance was in the range of 1–3%/l, adjusted to a respiratory rate of 30–50 breaths/min and respiratory gating was performed. Ophthalmic ointment was applied to protect the eyes from drying. Localisers were acquired in three spatial directions. A morphological gradient-echo sequence was acquired in transverse orientation with a repetition time of 15 ms, an echo time of 4.7 ms and a flip angle of 15°. For MT measurement, a three-dimensional zero echo time (ZTE) sequence was applied in this study as previously reported [11, 23]. Acquisition parameters of the MT measurements are summarised in Table 2.

**Table 2:** Acquisition parameters of the MT-prepulse and the ZTE sequence

Sequence parameter: MT-prepulse	
Shape	Gaussian-shaped
Duration (ms)	10
Nominal flip angle (°)	1000 or 3000
Bandwidth full-width-at-half-maximum (Hz)	274
Off-resonance frequencies (Hz)	1000, 2000, 3000, 4000, 6000, 8000, 10,000 and 15,000
Sequence parameter: ZTE readout parameters	
Length of the hard pulse (μs)	1
Flip angle (°)	3.9
Matrix size	160
Repetition time (ms)	1
Signal averages (n)	2
Isotropic resolution (mm)	0.31
Bandwidth (kHz)	200

### Post-processing and image analysis

Since background signal of animal bed and radiofrequency coil was observed, a subtraction approach using a D_2_O phantom was used as previously described [23] to eliminate the observed background signal by subtraction.

The values of MT ratio (MTR) were calculated with in-house software using the programming language Matlab (The Mathworks, Natick, MA, USA). MTR was calculated using the equation

$$ MTR=\frac{M_0-{M}_{sat}}{M_0} $$

where *M*_*0*_ means the signal intensity without MT prepulse and *M*_*sat*_ means the respective signal intensity with MT prepulse.

**Fig. 1:**
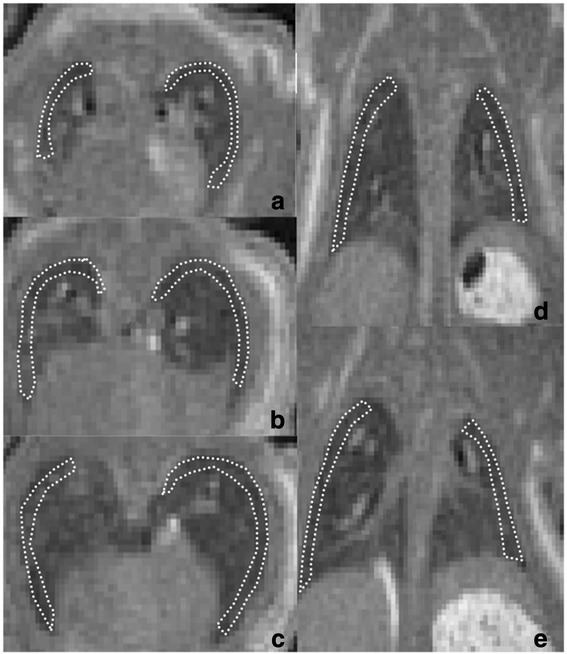
Example of ROI placement in axial orientation on an apical (**a**), central (**b**) and basal level (**c**), and in coronal orientation on dorsal (**d**) and ventral (**e**) levels

For MTR measurements, regions of interests (ROIs) were placed at the periphery of the transplanted and native lungs avoiding large vessels. ROI placement was systematically performed on a ventral, a dorsal, a basal, a medial and an apical part of the lung. An example of a typical ROI placement is shown in Fig. 1.

Two radiologists with 12 and four years of experience in diagnostic radiology assessed the morphological findings of the images in consensus reading for image quality, ventilated or infiltrated lungs, or pleural effusion. Sufficient image quality was defined as complete depiction of the lungs and the absence of any artifacts impeding the evaluation of the MTR maps. An infiltration was defined as an area of clearly higher signal intensity in the transplanted lung as compared to the contralateral side.

### Statistical analysis

The mean and standard deviation of MTR values were calculated for descriptive analysis and the Kolmogorov–Smirnov test was applied to test for normality. Using a repeated measurement analysis of variance, the mean values of all ROI of all off-resonance frequencies and flip angles of the transplanted and not transplanted lungs were compared to evaluate the following factors: localisation of ROI placement, off-resonance frequency; flip angle; and transplantation/no transplantation. After checking for normality using Kolmogorov–Smirnov test, Student’s t-test was performed for all flip angles and off-resonance frequencies for the following different groups: comparison A: the transplanted well-ventilated vs transplanted infiltrated lung tissue; comparison B: the transplanted lung with CAF (including well-ventilated and infiltrated lung tissue) vs the transplanted lung without CAF (including well-ventilated and infiltrated lung tissue); comparison C: the transplanted infiltrated lung tissue with CAF vs the transplanted infiltrated lung tissue without CAF. Bonferroni correction was applied to compensate for multiple comparisons. The software package SPSS 22.0 (IBM Corp., Somers, NY, USA) was used and a *p* value < 0.050 was considered as significant.

## Results

### Histological assessment

**Fig. 2:**
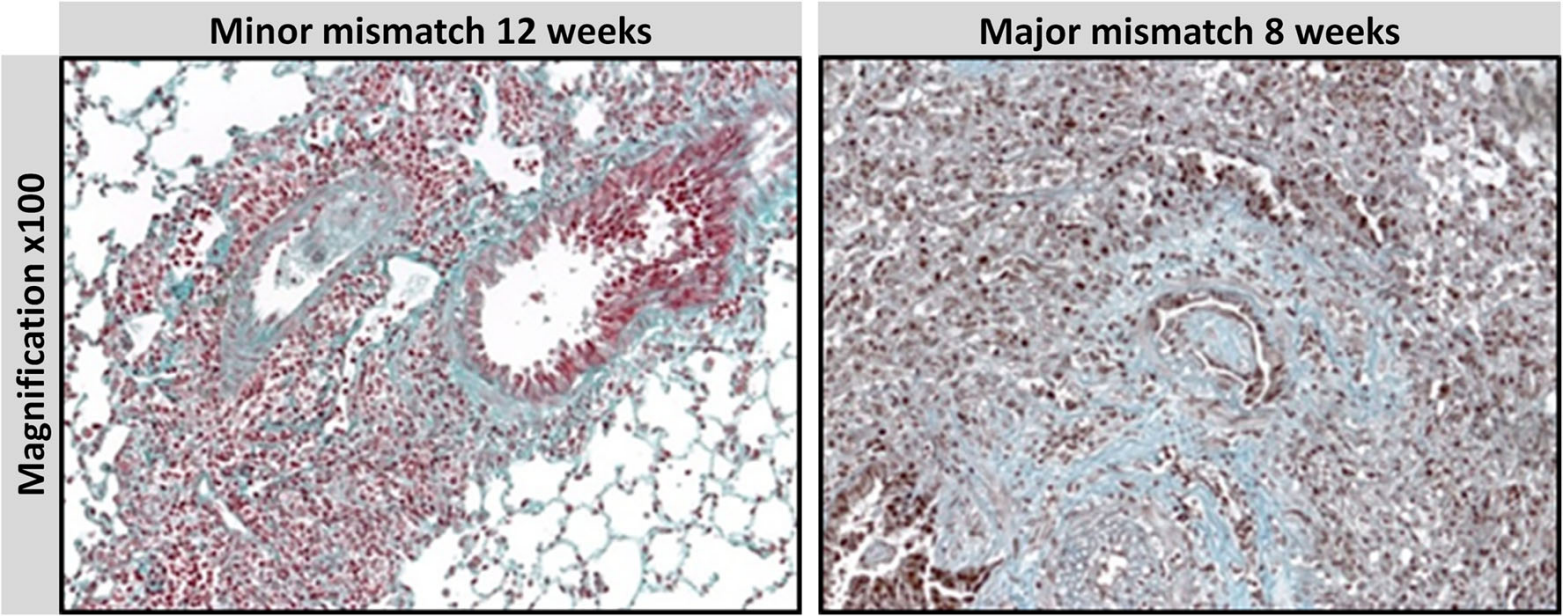
Representative histologic sections: collagen depositions can be seen in trichrome staining as cyan colour. Minor MHC mismatch combination (*left*) showed mild peri-airway fibrosis but no intraluminal fibrosis. In contrast, major MHC mismatch combination (*right*) showed fibrotic tissue around and inside airways, as well as in the parenchyma

Lung transplantation was successfully performed in all 24 recipient mice. Seven animals showed a pattern of CAF in the histologic sections while 17 showed no signs of CAF. In the haematoxylin and eosin staining signs of acute rejection were also observed, including the presence of mononuclear interstitial cell infiltrates and complete cuffing of the vessels. All mice that developed a CAF also showed signs of acute rejection: in two samples mild acute rejection and in five samples moderate to severe acute rejection according to ISHLT grades was observed. Table 1 gives an overview of the type of donor/recipient combination, the mean acute rejection score, number of CAF and the time between transplantation and explantation. Figure 2 shows representative sections of the histologic examination.

### Qualitative image evaluation

Some of the transplanted lungs appeared to be well-ventilated (*n* = 10) during the MRI examination and others were infiltrated (*n* = 14). The lung tissue that was infiltrated exhibited a CAF in six animals and no signs of CAF were present in eight animals. In none of the examined mice was an effusion observed.

**Fig. 3:**
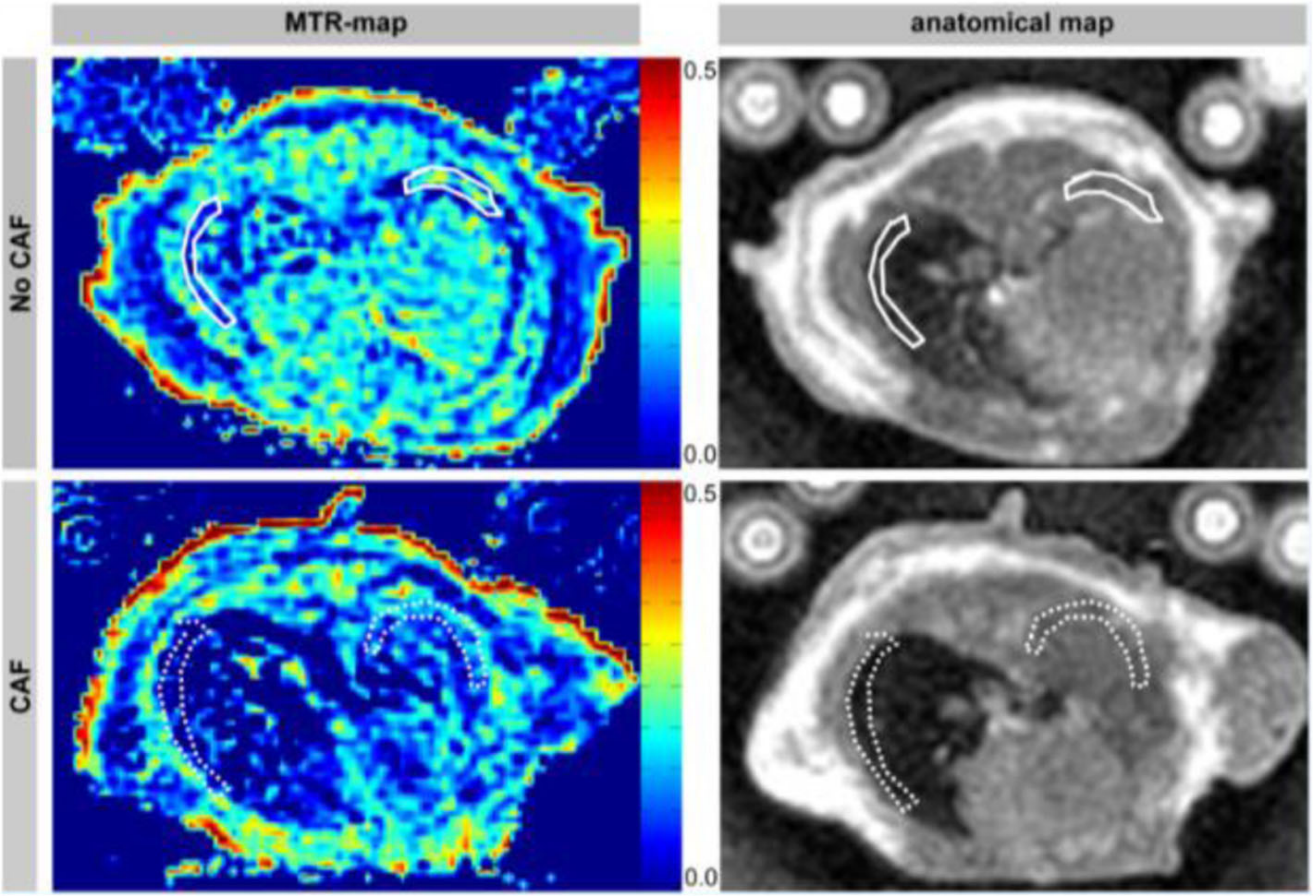
Examples of MTR *maps* (1000° flip angle and 4000 Hz off-resonance frequency) and anatomical images of infiltrated mouse lungs with and without CAF. On the anatomical maps one can see infiltration in the transplanted lungs of both mice with CAF (*dotted ROI* on the *right*) and without CAF (*solid line ROI* on the *right*). The non-transplanted side (ROIs on the *left*) of the anatomical map show low signal of the non-infiltrated lungs. MTR maps indicate CAF in the infiltrated lung: the infiltrated lungs without CAF show higher MTR values (solid line ROI on the *right*) than the infiltrated lungs with CAF (dotted ROI on the *right*). MTR values of the non-transplanted side are lower compared to the transplanted side of both mice (ROIs on the *left*)

The image quality of the MTR maps was sufficient and allowed the depiction of the non-transplanted and transplanted lungs. Figure 3 shows typical examples of MTR maps.

### Quantitative image evaluation (MTR measurements)

Multivariate analysis of variance showed no significant differences if the ROI was placed in a ventral, a dorsal, a basal, a medial and an apical part of the lung. (*p* = 0.342), thus all MTR values reported are an average of the five different ROIs. We observed significant differences for different off-resonance flip angles (*p* = 0.001) and off-resonance frequencies (*p* = 0.001). The MTR values were always higher at a 3000° flip angle compared to a 1000° flip angle. The highest MTR values were observed at a 3000° flip angle and a 1000-Hz off-resonance frequency.

Comparing the transplanted with the non-transplanted lungs, we observed significantly lower MTR in the not transplanted lungs (*p* = 0.026). No infiltration was observed in the non-transplanted lungs.

**Fig. 4:**
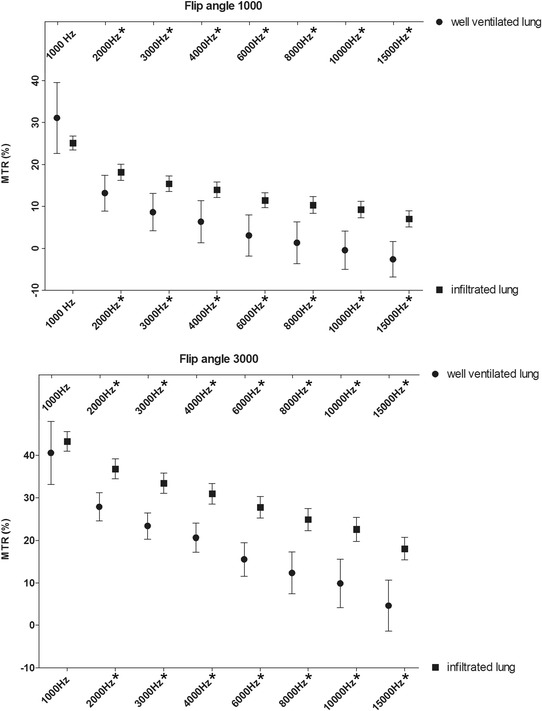
Comparison of the transplanted well-ventilated vs transplanted infiltrated lung tissue (comparison A). MTR mean values are depicted and *error bars* indicate the 95% confidence interval for all off-resonance frequencies and flip angles. *Asterisks* indicate comparisons with significant differences. In the following, we give the *p* values for a 1000° flip angle and all off-resonance frequencies (off-resonance frequency (Hz)/*p* value): 1000 Hz/*p* = 0.094, 2000 Hz/*p* = 0.011, 3000 Hz/*p* = 0.001, 4000 Hz/*p* = 0.001, 6000 Hz/*p* = 0.001, 8000 Hz/*p* = 0.001, 10,000 Hz/*p* = 0.001 and 15,000 Hz/*p* = 0.001. The *p* values for a 3000° flip angle and all off-resonance frequencies were as follows (off-resonance frequency (Hz)/*p* value): 1000 Hz/*p* = 0.473, 2000 Hz/*p* = 0.001, 3000 Hz/*p* = 0.001, 4000 Hz/*p* = 0.001, 6000 Hz/*p* = 0.001, 8000 Hz/*p* = 0.001, 10,000 Hz/*p* = 0.001 and 15,000 Hz/*p* = 0.001

**Fig. 5:**
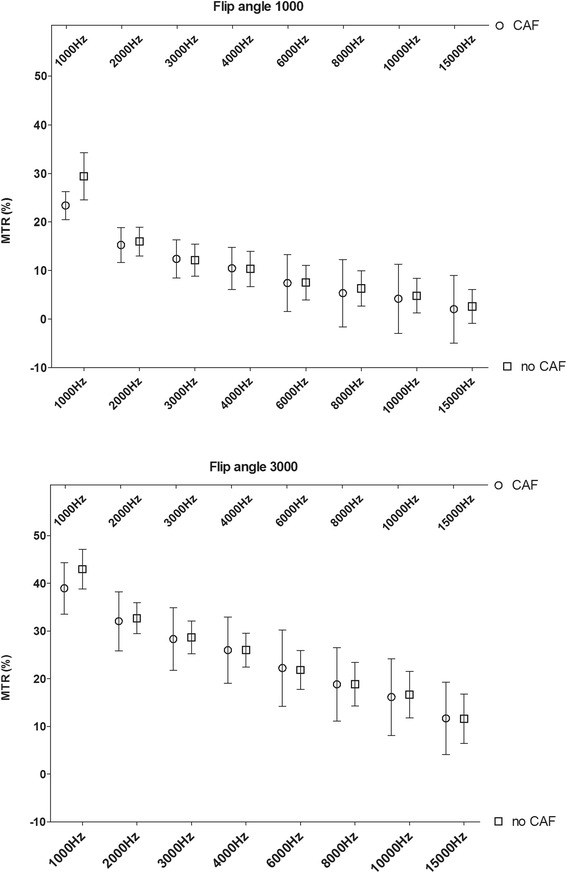
Comparison of the transplanted lung tissues with CAF (including well-ventilated and infiltrated lung tissue) vs the transplanted lung tissues without CAF (including well-ventilated and infiltrated lung tissue) (comparison B). MTR mean values are depicted and *error bars* indicate the 95% confidence interval for all off-resonance frequencies and flip angles. No significant differences were observed. In the following, we give the *p* values for a 1000° flip angle and all off-resonance frequencies (off-resonance frequency (Hz)/*p* value): 1000 Hz/*p* = 0.160, 2000 Hz/*p* = 0.931, 3000 Hz/*p* = 0.836, 4000 Hz/*p* = 0.872, 6000 Hz/*p* = 0.967, 8000 Hz/*p* = 0.795, 10,000 Hz/*p* = 0.936 and 15,000 Hz/*p* = 0.793. The *p* values for a 3000° flip angle and all off-resonance frequencies were as follows (off-resonance frequency (Hz)/*p* value): 1000 Hz/*p* = 0.282, 2000 Hz/*p* = 0.658, 3000 Hz/*p* = 0.364, 4000 Hz/*p* = 0.599, 6000 Hz/*p* = 0.535, 8000 Hz/*p* = 0.898, 10,000 Hz/*p* = 0.881 and 15,000 Hz/*p* = 0.680

**Fig. 6:**
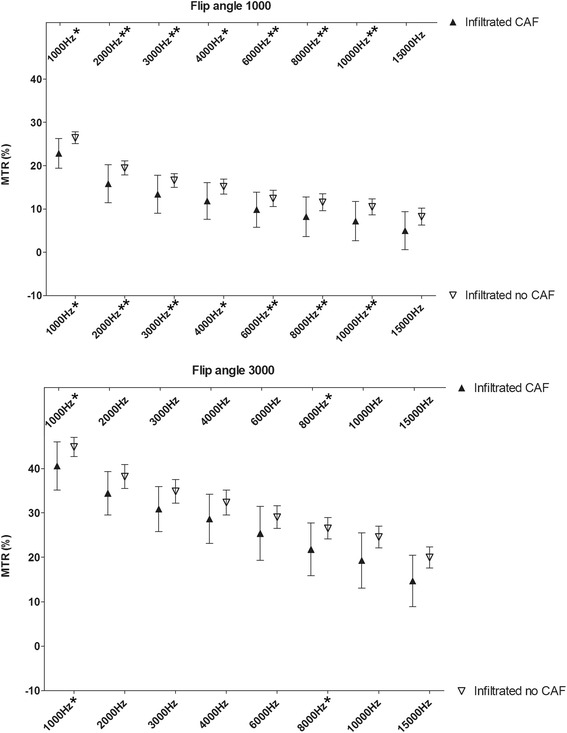
Comparison of the transplanted infiltrated lung tissue with CAF vs the transplanted infiltrated lung tissue without CAF (comparison C). MTR mean values are depicted and *error bars* indicate the 95% confidence interval for all off-resonance frequencies and flip angles. *Single asterisks* indicate significant *p* values; *two asterisks* indicate values close to significance (0.05 > *p* ≤ 0.09). In the following, we give the *p* values for a 1000° flip angle and all off-resonance frequencies (off-resonance frequency (Hz)/*p* value): 1000 Hz/*p* = 0.049, 2000 Hz/*p* = 0.057, 3000 Hz/*p* = 0.055, 4000 Hz/*p* = 0.050, 6000 Hz/*p* = 0.087, 8000 Hz/*p* = 0.058, 10,000 Hz/*p* = 0.074 and 15,000 Hz/*p* = 0.137. The *p* values for a 3000° flip angle and all off-resonance frequencies were as follows (off-resonance frequency (Hz)/*p* value): 1000 Hz/*p* = 0.053, 2000 Hz/*p* = 0.392, 3000 Hz/*p* = 0.739, 4000 Hz/*p* = 0.507, 6000 Hz/*p* = 0.350, 8000 Hz/*p* = 0.028, 10,000 Hz/*p* = 0.141 and 15,000 Hz/*p* = 0.190

Figures 4, 5 and 6 summarise the MTR values for different off-resonance frequencies and flip angles. Figure 4 depicts the MTR values of comparison A: the postoperatively well-ventilated vs the postoperatively infiltrated lung tissue. Well-ventilated lung tissue showed a significantly lower MTR for nearly all flip angles and off-resonance frequencies except for the lowest off-resonance frequency of 1000 Hz at both flip angles of 1000° and 3000°, respectively. Figure 5 shows comparison B: the transplanted lung with CAF (including well-ventilated and infiltrated lung tissue) vs the transplanted lung without CAF (including well-ventilated and infiltrated lung tissue). In this comparison, there were no significant differences for all flip angles and off-resonance frequencies. Figure 6 displays the results of comparison C: the transplanted infiltrated lung tissue with CAF vs the transplanted infiltrated lung tissue without CAF. The infiltrated lung without CAF exhibited higher MTR values compared to infiltrated lungs with CAF for all frequencies and flip angles; however, these differences were not always significant. This finding is further underlined by Fig. 3, which shows a typical example of such a MTR map. The decreased MTR of the infiltrated lung due to CAF can clearly be distinguished from the higher MTR in the infiltrated lungs without CAF.

## Discussion

In this study, we showed that lung tissue showing infiltration after lung transplantation could exhibit lower MT in the presence of CAF compared to infiltrated lung tissue without CAF, thereby providing a potential tool to assess the origin of pulmonary infiltration after lung transplantation. Moreover, we demonstrated that MT is increased in infiltrated lung tissue compared to normal appearing well-ventilated lung after lung transplantation, which may be attributed to the increased content of protein-rich substrates. During acute rejection, a variety of inflammatory cells release different proteins (e.g. different interleukins). During CAF, a fibrotic change of the tissue caused by collagen can be observed. In this study, we could see an MTR increase in acute rejection and an MTR decrease in CAF. Since MT is sensitive to changes in the interaction of ‘free’ and ‘bound’ protons, one reason might be that the different proteins associated with acute reaction and CAF cause a different composition of the ‘free’ and ‘bound’ water pool, possibly because of differences in their ability to build a solvation shell. As a consequence, different MTRs can be seen.

The depiction of lung tissue is known to be challenging due to the short T2* relaxation time of approximately 770 μs of lung tissue at 4.7 T. We therefore used a ZTE data readout in this study as previously reported [11, 23]. This is the first study using ZTE imaging for the depiction of chronic rejection of lung tissue. With this approach, the image quality of the MTR maps was sufficient and allowed the depiction of the non-transplanted and transplanted lungs in vivo with sufficient signal-to-noise ratio for meaningful MTR quantification.

We showed that lung transplantation itself caused significant changes in MTR values, where lower MTR in the non-transplanted lungs were observed. These differences are likely caused by a higher protein content in the infiltrated lung tissue, which leads to a significantly higher MT due to the interaction of the macromolecular spin pool with the water spin pool. This observation is in line with findings in an acute rejection lung transplantation model [24], in which a higher MTR was observed in lung exhibiting acute rejection. Conversely, well-ventilated lung tissue showed a significantly lower MTR for nearly all flip angles and off-resonance frequencies compared to infiltrated lung except for an off-resonance frequency of 1000 Hz. This effect may be attributed to direct saturation, as it is known that direct saturation effects may be present at low off-resonance frequencies [11].

There were no significant differences when comparing the transplanted lungs with CAF and without CAF without taking differences in ventilation or infiltration of the lung tissue into account. However, MTR allowed for detecting CAF in the infiltrated lung. MTR is sensitive to alterations in the macromolecule composition. During CAF, a thickening of the walls of the airways caused by collagen can be observed; therefore, one can hypothesise that the difference in MTR is caused by the interaction of collagen and the ‘free-water-protons’, especially when sufficiently high off-resonance frequencies were applied to preclude relevant direct saturation effects.

This ability of MTR contrast might widen the spectrum of diagnosing CAF, since differential diagnosis of high-resolution CT scans showing pathological findings after lung transplantation is still challenging. The MRI MT technique investigated here may provide additional information for the detection of CAF after lung transplantation.

Our study has limitations to be considered. First, the sample size of seven animals that finally developed CAF was small. However, due to ethical considerations according to the ‘3R’ principle (replacement, reduction and refinement) in animal research [25], we considered this sample size to be sufficient to demonstrate the principle value of MTR for the detection of CAF. Unfortunately, the majority of the lungs with CAF exhibited significant infiltration; therefore, with our data we cannot prove that CAF can also be detected in well-ventilated lungs. Second, the patterns of major and minor mismatch in MHC combinations of the donor and the graft mice was not completely homogeneous in this study. The reason for this variation was that we experienced strong difficulties in reliably inducing CAF in our model. Therefore, we tested several different combinations of donor and recipient mouse strains as well as the time span for CAF development. We kept all mice in the study to keep the number of animals as low as possible. Third, in our animal model, all animals with CAF also showed acute rejection. However, the MTR differences between lungs with and without CAF were higher than in acute rejection alone, as previously published [24]. The findings are, however, not directly comparable due to the differences in the animal models.

In conclusion, we showed that MT measurement might serve as a non-invasive tool for the detection of CAF in infiltrated lung tissue after lung transplantation. Differences in MT observed between lungs exhibiting CAF and those exhibiting acute rejection alone may be attributed to differences in the macromolecular composition of the tissues. The potential value of MTR in this setting on humans warrants future clinical investigations.

## Abbreviations

CAF: Chronic airway fibrosis
ISHLT: International Society of Heart and Lung Transplantation
MHC: Major histocompatibility complex
MRI: Magnetic resonance imaging
MT: Magnetisation transfer
MTR: Magnetisation transfer ratio
ROI: Regions of interest
ZTE: Zero echo time
